# Supplementary material for: How career adaptability affects entrepreneurial intention: The chain-mediated roles of entrepreneurial passion and self-efficacy
Source: PLoS One. 2025 Sep 2;20(9):e0329651. doi: 10.1371/journal.pone.0329651 (PMC12404465; doi:10.1371/journal.pone.0329651)
Supplement: Attachment — Submitted filename: Comments on the Manuscript PLOS One.docx [file pone.0329651.s001.docx]

**Abstract**

The abstract captures the essence of the research, summarizing the findings and implications succinctly.

**Suggestions**:

The term "chain-mediated effects" might be unfamiliar to some readers. Consider briefly defining it in lay terms.

The phrase "significantly positively correlated" is repeated. Instead, emphasize the practical significance or contribution of the findings.

Include a sentence highlighting the broader implications for entrepreneurship education globally, beyond the Chinese context.

**Introduction**

The introduction frames the research problem well, linking entrepreneurial intention (EI) to career adaptability (CA) using both theoretical and practical justifications. The rationale for including entrepreneurial passion (EP) and entrepreneurial self-efficacy (ESE) as mediators is strong and supported by relevant literature.

**Suggestions**:

Discuss the global relevance of CA in entrepreneurship. Does CA influence EI differently across cultural or economic contexts? This could broaden the appeal of your research.

Strengthen the argument about why previous studies on the direct CA-to-EI link yielded inconsistent results. Delve into potential gaps in methodology or theoretical frameworks.

Expand on how the study contributes to resolving these inconsistencies, particularly through the novel use of **Social Cognitive Theory** (SCT) and chain mediation.

**Methodology**

The selection of well-validated scales ensures reliability, and the large sample size enhances the robustness of the findings. Ethical considerations, including informed consent and institutional review board approval, are adequately addressed.

**Suggestions**:

Clarify the reasoning behind selecting only students from Zhejiang Province and the potential implications of this regional focus. Could this limit generalizability?

The high response rate (75.04%) is commendable, but discuss how potential biases (e.g., non-responders) were mitigated.

While Model 6 of the PROCESS macro is appropriate, explain why it was chosen over alternative models for testing mediation.

**Results**

Results are presented logically, with clear links to hypotheses. Tables and figures are used effectively to illustrate findings.

**Suggestions**:

Highlight the practical significance of the findings, beyond statistical significance. For instance, what does a 71.6% mediation effect of EP mean for entrepreneurship programs?

Discuss unexpected or non-significant results in more depth. For example, why was CA’s direct effect on EI non-significant, and how does this align with SCT?

Explore subgroup differences (e.g., gender, family entrepreneurial background) more thoroughly. Are there nuanced trends that could add depth to the analysis?

**Discussion**

The discussion effectively integrates findings with **Social Cognitive Theory** (SCT) and other theoretical frameworks, demonstrating a strong theoretical grounding. It highlights the mediating roles of EP and ESE as crucial pathways between CA and EI.

**Suggestions**:

Compare your findings with prior studies in more detail. How do the chain-mediated effects of EP and ESE in your study differ from or align with previous research?

Offer a critical evaluation of why SCT provides a better framework for understanding these relationships than other potential theories (e.g., Theory of Planned Behavior).

Discuss broader implications of the findings. For instance, could interventions to boost CA also enhance non-entrepreneurial career outcomes?

**Implications**

The practical suggestions for enhancing entrepreneurship education are timely and actionable.

**Suggestions**:

Provide specific examples of how universities can operationalize these insights. For instance, how can courses like "Career Life Planning" be modified to explicitly include elements that boost EP and ESE?

Discuss policy-level implications. How can governments or educational policymakers leverage your findings to improve national entrepreneurship outcomes?

Suggest tools or strategies for measuring improvements in EP and ESE over time, linking these to interventions aimed at boosting CA.

**Limitations and Future Research**

The study acknowledges key limitations, including its cross-sectional design and focus on university students in a specific region.

**Suggestions**:

The cross-sectional nature of the study is a significant limitation. Discuss how a longitudinal or experimental design could better establish causality.

Address the potential for cultural bias, given the focus on Chinese students. Would the findings hold in countries with less emphasis on entrepreneurship education?

Highlight the need for future research to examine other mediators (e.g., personality traits or external factors like access to funding) that could influence the CA-to-EI relationship.

**Conclusion**

The conclusion succinctly summarizes the findings and reiterates their importance.

**Suggestions**:

Avoid simply repeating the discussion points. Instead, use the conclusion to present a visionary outlook for the field. For example, how might this research transform the way entrepreneurship education is delivered?

Suggest a concrete next step for researchers or educators based on your findings.

**Figures and Tables**

Figures and tables are well-constructed and enhance comprehension.

**Suggestions**:

For Table 3, explicitly interpret the percentage contributions of direct and indirect effects to the total effect in the text.

**References**

The references are up-to-date and cover a broad range of relevant studies.

**Suggestions**:

Ensure consistency in citation formatting (e.g., journal names in italics).

Discuss any gaps in the literature that your study addresses explicitly.

These deeper comments aim to guide further refinement of the manuscript, ensuring both theoretical and practical contributions are maximized.
