# Supplementary material for: How career adaptability affects entrepreneurial intention: The chain-mediated roles of entrepreneurial passion and self-efficacy
Source: PLoS One. 2025 Sep 2;20(9):e0329651. doi: 10.1371/journal.pone.0329651 (PMC12404465; doi:10.1371/journal.pone.0329651)
Supplement: Attachment — Submitted filename: [1.27]Response to Reviewers.docx [file pone.0329651.s002.docx]

Dear Editor:

It is our big honor to have you be our editor, and we appreciate your attention and recognition for our manuscript. Many thanks to reviewers for giving us a lot of valuable suggestions. Based on the suggestions of the reviewers, we have made a lot of revisions to the manuscript, and we are confident to submit a high-quality manuscript. Below are our responses based on the comments of the reviewers, thank you again.

**To** **reviewers 1:**

Dear reviewer 1:

Thank you very much for your time involved in reviewing the manuscript and your comments have further improved the quality of the manuscript. We have carefully reviewed the comments and revised the manuscript accordingly. Hope the explanation has fully addressed all of your concerns. Point-by-point responses to reviewers are as follows:

**Q1:Abstract**

The abstract captures the essence of the research, summarizing the findings and implications succinctly.

**Suggestions:**

The term "chain-mediated effects" might be unfamiliar to some readers. Consider briefly defining it in lay terms.

The phrase "significantly positively correlated" is repeated. Instead, emphasize the practical significance or contribution of the findings.

Include a sentence highlighting the broader implications for entrepreneurship education globally, beyond the Chinese context.

***Reply1:***Thank you for your valuable comments. We have rewritten the summary at your suggestion.

We replaced "significantly positively correlated" with "interrelated in ways that suggest meaningful pathways for enhancing entrepreneurial outcomes", avoiding repetition and emphasizing the value of the findings to the actual situation. We have also added a sentence of "While focused on Chinese university students, the implications extend globally..." to guide the significance of expanded research for global entrepreneurship education. For details, please see lines 48 - 70.

In addition, we also help the reader understand in the introduction by explaining that "chain-mediated" means that "a factor sequentially affects the final result through two or more continuous mediating variables", Please see for details lines 118-124. This idea is also reflected in the abstract, "This study examined how entrepreneurial self-efficacy and entrepreneurial passion jointly mediate the relationship between career adaptability and entrepreneurial intention." please see lines 49-51.

**Q2:Introduction**

The introduction frames the research problem well, linking entrepreneurial intention (EI) to career adaptability (CA) using both theoretical and practical justifications. The rationale for including entrepreneurial passion (EP) and entrepreneurial self-efficacy (ESE) as mediators is strong and supported by relevant literature.

**Suggestions:**

Discuss the global relevance of CA in entrepreneurship. Does CA influence EI differently across cultural or economic contexts? This could broaden the appeal of your research.

Strengthen the argument about why previous studies on the direct CA-to-EI link yielded inconsistent results. Delve into potential gaps in methodology or theoretical frameworks.

Expand on how the study contributes to resolving these inconsistencies, particularly through the novel use of Social Cognitive Theory (SCT) and chain mediation.

***Reply2:***Thank you for your valuable comments. We have rewritten the introduction, following your suggestion. The new version explores the global relevance of CA in entrepreneurship, see lines 87-91.

Results that explore the inconsistent direct effect of the existing literature CA-EI. The inconsistent results of studies on the relationship between CA and EI might be due to differences in theoretical frameworks, or neglect of key mediating variables [10], or different cultural or economic backgrounds [66]. For details, please see L93-95.

We explore the use of SCT as the theoretical basis, the key mediation variables and the chain mediation model, and the cultural background in China. Explore how to extend the CA-EI studies to address inconsistent arguments in previous studies. Please see lines 74-136 for further details. In addition, we have added some new literature.

**Q3:Methodology**

The selection of well-validated scales ensures reliability, and the large sample size enhances the robustness of the findings. Ethical considerations, including informed consent and institutional review board approval, are adequately addressed.

**Suggestions:**

Clarify the reasoning behind selecting only students from Zhejiang Province and the potential implications of this regional focus. Could this limit generalizability?

The high response rate (75.04%) is commendable, but discuss how potential biases (e.g., non-responders) were mitigated.

While Model 6 of the PROCESS macro is appropriate, explain why it was chosen over alternative models for testing mediation.

***Reply:***Thank you for your valuable comments. Your suggestions are of great help to improve the paper. Here are the modifications and explanations we have made, and we will reply one by one, as follows:

3.1 Clarify the reasoning behind selecting only students from Zhejiang Province and the potential implications of this regional focus. Could this limit generalizability?

***Reply 3.1:*** This study mainly focuses on college students in Zhejiang Province, the reason is that Zhejiang Province, as a region with more developed entrepreneurship education, has a high level of economic development and a strong atmosphere of innovation and entrepreneurship. In addition, the three schools selected in this study were designated as vocational and entrepreneurship education model schools by the Ministry of Education of China, and these schools all offered "career planning" courses. All participants had completed the course and demonstrated high occupational resilience, which provided an ideal sample for the study (detailed in lines 300-306). However, sample selection by rationalization may impose limitations on the broad applicability of the study. Therefore, future studies could extend the survey to other regions, especially those with different cultural and economic backgrounds, to test the universality of the findings. This is detailed in limitations and future studies (see lines 683-700).

3.2 The high response rate (75.04%) is commendable, but discuss how potential biases (e.g., non-responders) were mitigated.

***Reply 3.2:*** This study distributed the questionnaires to students after obtaining official and student consent by contacting the university's entrepreneurship guidance departments. The online survey link was distributed randomly over the internet to college students. Out of 1350 questionnaires sent, 1,013 valid responses were received, yielding a response rate of 75.04%. To reduce potential bias (for example, non-respondents may hold different opinions or characteristics), the study adopted random distribution and anonymous answers during the data collection process. In addition, a preliminary examination of the non-respondent sample found that the differences between non-respondents and valid respondents in key variables such as gender, major, and whether family members have entrepreneurial experience were small, further reducing the potential risk of bias (see lines 311-317).

3.3 While Model 6 of the PROCESS macro is appropriate, explain why it was chosen over alternative models for testing mediation.

***Reply 3.3:*** We supplemented the article and also added some literature. Content is as follows:

Secondly, the chain mediation effects were examined using Pearson correlation analysis and the PROCESS plug-in for SPSS. The PROCESS plug-in is used with a combination of moderating and mediation analyses based on path analysis [57]. This study uses Model 6 of the PROCESS macro plug-in of SPSS as the analysis framework, with career adaptability (CA) set as the independent variable, entrepreneurial passion (EP) and entrepreneurial self-efficacy (ESE) set as chain mediating variables, and entrepreneurial intention (EI) as the dependent variable. The reason for choosing Model 6 is that this model can test the chain effects of multiple mediating variables and can systematically analyze the multi-path indirect effects of CA on EI through EP and ESE [57]. In contrast, other models (such as Model 4 or Model 7) cannot capture the relationship between multiple mediating variables at the same time, and are therefore not suitable for hypothesis verification in this study [57]. Moreover, most of the current empirical studies on chain mediation models have adopted this data analysis method [67-68]. (see lines 385-395)

**Q4:Results**

Results are presented logically, with clear links to hypotheses. Tables and figures are used effectively to illustrate findings.

**Suggestions:**

Highlight the practical significance of the findings, beyond statistical significance. For instance, what does a 71.6% mediation effect of EP mean for entrepreneurship programs?

Discuss unexpected or non-significant results in more depth. For example, why was CA’s direct effect on EI non-significant, and how does this align with SCT?

Explore subgroup differences (e.g., gender, family entrepreneurial background) more thoroughly. Are there nuanced trends that could add depth to the analysis?

***Reply:*** Thank you for your valuable comments. Your suggestions are of great help to improve the paper. Here are the modifications and explanations we have made, and we will reply one by one, as follows:

4.1 Highlight the practical significance of the findings, beyond statistical significance. For instance, what does a 71.6% mediation effect of EP mean for entrepreneurship programs?

***Reply 4.1:*** We have added the part of "6.2 Practical Implications" to fully explore the practical significance of statistical results on university management. Please see lines 605- 669.

What does a 71.6% mediation effect of EP mean for entrepreneurship programs？For this problem, We answered in the article: " EP accounts for the majority of the total effect (71.6%), which shows that entrepreneurial passion plays a very key role in the impact of career adaptability on entrepreneurial intention (please see lines 449 - 452). The practical significance of this part, We have added, specifically to the article, Please see lines 617-623 as follows: “Given that EP accounts for the vast majority of the total effect, this finding highlights the critical role of emotional factors (such as EP) in entrepreneurship education. Universities can focus on content that promotes the development of EP to facilitate the transformation from CA to EI. For instance, inviting entrepreneurs to share their success stories or having instructors demonstrate their identification with the entrepreneurial identity and express their EP can inspire students.”

4.2 Discuss unexpected or non-significant results in more depth. For example, why was CA’s direct effect on EI non-significant, and how does this align with SCT?

***Reply 4.2:***We rewrite the discussion sections, provided a deeper discussion of the results, and discussed unexpected or non-significant results (see lines 470-565). For example, it further explains, "why was CA’s direct effect on EI non-significant, and how does this align with SCT?"This question. Please see lines 482-510.

4.3 Explore subgroup differences (e.g., gender, family entrepreneurial background) more thoroughly. Are there nuanced trends that could add depth to the analysis?

***Reply 4.3:***A subsection of "4.2 Difference Analysis" is added to this chapter to Explore subgroup differences (e.g., gender, family entrepreneurial background) more thoroughly.See lines 419-431. The added analysis and practical implications are discussed according to the data results. Please see lines 634-641。

**Q5:Discussion**

The discussion effectively integrates findings with Social Cognitive Theory (SCT) and other theoretical frameworks, demonstrating a strong theoretical grounding. It highlights the mediating roles of EP and ESE as crucial pathways between CA and EI.

**Suggestions:**

Compare your findings with prior studies in more detail. How do the chain-mediated effects of EP and ESE in your study differ from or align with previous research?

Offer a critical evaluation of why SCT provides a better framework for understanding these relationships than other potential theories (e.g., Theory of Planned Behavior).

Discuss broader implications of the findings. For instance, could interventions to boost CA also enhance non-entrepreneurial career outcomes?

***Reply:***Your comments are very professional and we appreciate them. Here are the modifications and explanations we have made, and we will reply one by one, as follows:

5.1 Compare your findings with prior studies in more detail. How do the chain-mediated effects of EP and ESE in your study differ from or align with previous research?

***Reply5.1:*** According to your Suggestions，We rewrite this part of Discussion, see lines 470-565. We also specifically added Explain about how do the chain-mediated effects of EP and ESE in your study differ from or align with previous research. Please see lines 482-510 and lines 541-565.

5.2 Offer a critical evaluation of why SCT provides a better framework for understanding these relationships than other potential theories (e.g., Theory of Planned Behavior).

***Reply5.2:*** We added this part to explain that SCT provides a better framework for understanding these relationships than other potential theories (e.g., Theory of Planned Behavior). Please see lines 592-604.

5.3 Discuss broader implications of the findings. For instance, could interventions to boost CA also enhance non-entrepreneurial career outcomes?

***Reply5.3:*** We added this section to discuss the broader implications of these findings, see lines 566–669. Specifically discussing whether interventions to promote the development of CA can also improve non-entrepreneurial career outcomes, see lines 663 – 669.

**Q6:Implications**

The practical suggestions for enhancing entrepreneurship education are timely and actionable.

**Suggestions:**

Provide specific examples of how universities can operationalize these insights. For instance, how can courses like "Career Life Planning" be modified to explicitly include elements that boost EP and ESE?

Discuss policy-level implications. How can governments or educational policymakers leverage your findings to improve national entrepreneurship outcomes?

Suggest tools or strategies for measuring improvements in EP and ESE over time, linking these to interventions aimed at boosting CA.

***Reply:***Your comments are very professional and we appreciate them. We have rewritten the chapter, adding the suggestions mentioned above. Please see lines 566-669

**Q7:Limitations and Future Research**

The study acknowledges key limitations, including its cross-sectional design and focus on university students in a specific region.

**Suggestions:**

The cross-sectional nature of the study is a significant limitation. Discuss how a longitudinal or experimental design could better establish causality.

Address the potential for cultural bias, given the focus on Chinese students. Would the findings hold in countries with less emphasis on entrepreneurship education?

Highlight the need for future research to examine other mediators (e.g., personality traits or external factors like access to funding) that could influence the CA-to-EI relationship.

***Reply:***Your comments are very professional and we appreciate them. We have rewritten the chapter, adding the suggestions mentioned above. Please see lines 670-707。

**Q8:**Conclusion

The conclusion succinctly summarizes the findings and reiterates their importance.

Suggestions:

Avoid simply repeating the discussion points. Instead, use the conclusion to present a visionary outlook for the field. For example, how might this research transform the way entrepreneurship education is delivered?

Suggest a concrete next step for researchers or educators based on your findings.

***Reply:*** Your comments are very professional and we appreciate them. We have rewritten the chapter, adding the suggestions mentioned above. Please see lines 709-727.

**Q9:Figures and Tables**

Figures and tables are well-constructed and enhance comprehension.

**Suggestions:**

For Table 3, explicitly interpret the percentage contributions of direct and indirect effects to the total effect in the text.

***Reply:*** Thank you for your Suggestions. We have added this part of the explanation, please see lines 446-464.

**Q10:References**

The references are up-to-date and cover a broad range of relevant studies.

**Suggestions:**

Ensure consistency in citation formatting (e.g., journal names in italics).

Discuss any gaps in the literature that your study addresses explicitly

***Reply:***Thank you for your Suggestions, the reference format has been fully updated. We rewrite the introduction, discussion, and meaning sections, explicitly identifying any gaps in the literature. Further refine the article to maximize the contribution of theory and practice.

**To reviewers 2:**

Dear reviewer 2:

Thank you very much for your time involved in reviewing the manuscript and your comments have further improved the quality of the manuscript. We have carefully reviewed the comments and revised the manuscript accordingly. Hope the explanation has fully addressed all of your concerns. Point-by-point responses to reviewers are as follows：

**Q1:**Explain chain-mediated effect in the introduction and briefly distinguish this with mediation.

***Reply:***Thank you for your valuable comments. Following your suggestion, we have revised the introduction to add this part emphatically. Please see lines 118-124, content is as follows:

The mediation effect focuses on a single mediation variable and explains how the independent variable affects the dependent variable[57]. Chain mediation refereed to "a factor that affects the final result in sequence through two or more continuous mediating variables [57]. And shows how independent variables progressively influence the dependent variable through a series of mediating variables [57]. Chain mediation effects can provide more complex models of relationships, revealing how multiple mental processes or mechanisms work together [57].

**Q2:**It is true that career adaptability as a variable and its link with EI are not explored much in the field of student entrepreneurship across the globe and studying only a region in this study may be biased. Author should either add it as a limitation or compare the results with other studies to present the study’s outcome more prominently.

***Reply:***Thank you for your valuable comments. We rewrite the part of limitations and future research directions to highlight this proposal. Please see lines 683-700.

**Q3:**High number of female students may also bring the bias in the study. Authors may also include the descriptive statistics of the data to make it more interesting. (eg. How many girls’ students are from business background family.)

***Reply:***Thank you for the reminder. We acknowledge that the large number of girls in the sample in this study may introduce the bias in the study. However, there is a large proportion of women, which is relatively common in some universities in China. This is consistent with the gender distribution of the target population, and thus partly reflects actual social phenomena. In the empirical study, we also regarded gender as a control variable to further exclude the influence of gender on entrepreneurial intention. In addition, we added the normality analysis for all continuous variables, which met the criteria of less than 2 and kurtosis less than 7 in the absolute value, and the data were normally distributed. This provides a prerequisite for the validity of the subsequent data analysis. See lines 399-404 for specific modification.

We made descriptive statistics on the data following your recommendation and found 292 female family members have entrepreneurial experience. We added this in the article, please see lines 321-324. Content is as follows:

Among them, 80 male family members have entrepreneurial experience, while 123 male family members do not have entrepreneurial experience; 292 female family members have entrepreneurial experience, while 518 female family members do not have entrepreneurial experience.

**Q4:**Authors should check and explain if majority of the non-responders are from only one gender or any specific academic background.

***Reply:***Thank you for your valuable comments. The interpretation of this section has been added to the article, see lines 311-317. Content is as follows:

To reduce potential bias (for example, non-respondents may hold different opinions or characteristics), the study adopted random distribution and anonymous answers during the data collection process. In addition, a preliminary examination of the non-respondent sample found that the differences between non-respondents and valid respondents in key variables such as gender, major, and whether family members have entrepreneurial experience were small, further reducing the potential risk of bias.

**Q5:** Explain the reason behind the adoption of Pearson correlation analysis and the PROCESS plug-in for SPSS chain to examine the mediation effects over the other available alternatives.

***Reply:***Thank you for your valuable comments. The reason why we used Pearson's correlation analysis is that we need to determine whether continuous variables are related before further regression analysis. Our reason for using PROCESS has been added in the text, please see lines 385-395. Content is as follows:

Secondly, the chain mediation effects were examined using Pearson correlation analysis and the PROCESS plug-in for SPSS. The PROCESS plug-in is used with a combination of moderating and mediation analyses based on path analysis [57]. This study uses Model 6 of the PROCESS macro plug-in of SPSS as the analysis framework, with career adaptability (CA) set as the independent variable, entrepreneurial passion (EP) and entrepreneurial self-efficacy (ESE) set as chain mediating variables, and entrepreneurial intention (EI) as the dependent variable. The reason for choosing Model 6 is that this model can test the chain effects of multiple mediating variables and can systematically analyze the multi-path indirect effects of CA on EI through EP and ESE [57]. In contrast, other models (such as Model 4 or Model 7) cannot capture the relationship between multiple mediating variables at the same time, and are therefore not suitable for hypothesis verification in this study [57]. Moreover, most of the current empirical studies on chain mediation models have adopted this data analysis method [67-68].

**Q6:** Results are explained theoretically only in numbers. Kindly explain the interpretation and implication of these results especially non-significant in the light of social cognitive theory. Authors may add a “future research” section also and request the future researchers to explore the insignificant relation in other parts if the world.

***Reply:***Thank you for your valuable comments. Following your suggestion, we have rewritten the discussion, lmplications, limitations and future research section (lines 470-707). In the discussion and lmplications, focus on the interpretation and implication of these results (lines 470-669), especially the implication of social cognitive theory (lines 496-510; lines 534-540; lines 551-565 and lines 586-604). We have added a "Limitations and Future Research Directions" section also and request the future researchers to explore the insignificant relation in other parts if the world. Please see lines 683-700.

***Q7:*** Either in the discussion or in the introduction, Authors should add why they choose SCT over TPV or other alternative theories for this study.

***Reply:***Thank you for your valuable comments. We have added in the discussion the reasons for choosing SCT over TPV or other alternative theories of this study. Please see lines 592-604.
