# Supplementary material for: How career adaptability affects entrepreneurial intention: The chain-mediated roles of entrepreneurial passion and self-efficacy
Source: PLoS One. 2025 Sep 2;20(9):e0329651. doi: 10.1371/journal.pone.0329651 (PMC12404465; doi:10.1371/journal.pone.0329651)
Supplement: Attachment — Submitted filename: [2025.7.1]Response to Reviewers.docx [file pone.0329651.s003.docx]

Dear Editor:

It is our big honor to have you be our editor, and we appreciate your attention and recognition for our manuscript. Many thanks to reviewers for giving us a lot of valuable suggestions. Based on the suggestions of the reviewers, we have made a lot of revisions to the manuscript, and we are confident to submit a high-quality manuscript. Below are our responses based on the comments of the reviewers, thank you again.

**To** **reviewers 3:**

Dear reviewer 3:

Thank you very much for your time involved in reviewing the manuscript and your comments have further improved the quality of the manuscript. We have carefully reviewed the comments and revised the manuscript accordingly. Hope the explanation has fully addressed all of your concerns. Point-by-point responses to reviewers are as follows:

**Q1:** In Section 1. “Introduction”

Suggestions:

- Consider more global context early on (e.g., mention entrepreneurship education trends worldwide, not just China, before narrowing down).

***Reply1:***Thank you for your valuable comments. We have rewritten this section of the Introduction as per your suggestion. Please review lines 74-145. In particular, we have highlighted the global trends in entrepreneurship education before narrowing our focus; please refer to lines 74-89.

**Q2:** In Section 2. “Literature review and research hypothesis”

- The authors do not convincingly establish the novelty of their research. While the CA–EI link is not fully resolved in the literature, the manuscript lacks a systematic mapping or synthesis of the existing studies to position the current work.

- Applying Social Cognitive Theory (SCT) is a conventional approach in entrepreneurship research and does not, in itself, constitute theoretical innovation.

- The hypotheses are listed without strong theoretical justification between each link, especially H4.

***Reply2:***Thank you for your valuable comments. We have incorporated additional literature to bolster the persuasiveness of this section; please refer to lines 151-314. Specifically, we have revised the portion pertaining to Hypothesis H4 to reinforce its theoretical underpinnings; please refer to lines 245-303.

**Q3:**In Section 3.2 “Participants and Procedures”

- **Q3.1:**Gender imbalance is extreme (80% female), yet the manuscript lacks analysis of how this might affect the generalizability of findings.

***Reply3.1:***Thank you for your valuable comments. To assess the potential impact of gender bias, we have conducted the following supplementary analysis:
1. Control Variable Test

After including gender as a covariate in the PROCESS model, the path coefficients exhibited only minor alterations (for instance, the total effect of career adaptability on entrepreneurial intention shifted from β=0.524 to β=0.516, a change of Δβ=0.8%), suggesting that the gender variable did not significantly affect the relationship between the primary variables (refer to Tables 1 and 2 for further details).

Table 1: Without controlling for gender

| The regression equation | |  | Overall fitting index | |  | Significance of regression coefficient | |
| --- | --- | --- | --- | --- | --- | --- | --- |
| Outcome equation | Predictors |  | *R^2^* | *F* |  | *β* | *t* |
| EI | CA |  | 0.304 | 146.94^***^ |  | 0.524 | 19.546^***^ |
|  | Major（HSS=0） |  |  |  |  | 0.051 | 1.924 |
|  | EEFM/NEEFM（NEEFM=0） |  |  |  |  | 0.086 | 3.193^**^ |

*Note: β* means standardized coefficient; **p*<0.05; ***p*<0.01; ****p*<0.001; EEFM means entrepreneurial experience of family members; NEEFM means no entrepreneurial experience of family members; HSS means major in Humanities and social sciences.

Table 2 Control Gender

| The regression equation | |  | Overall fitting index | |  | Significance of regression coefficient | |
| --- | --- | --- | --- | --- | --- | --- | --- |
| Outcome equation | Predictors |  | *R^2^* | *F* |  | *β* | *t* |
| EI | CA |  | 0.314 | 115.55^***^ |  | 0.516 | 19.334^***^ |
|  | Gender（female=0） |  |  |  |  | 0.102 | 3.896^***^ |
|  | Major（HSS=0） |  |  |  |  | 0.053 | 2.026^*^ |
|  | EEFM/NEEFM（NEEFM=0） |  |  |  |  | 0.085 | 3.172^**^ |

*Note: β* means standardized coefficient; **p*<0.05; ***p*<0.01; ****p*<0.001; EEFM means entrepreneurial experience of family members; NEEFM means no entrepreneurial experience of family members; HSS means major in Humanities and social sciences.

2. Multiple Group Comparison Analysis

Despite the limited sample size of male participants (n=203), we conducted gender-based comparisons of the final model using permutation tests (5000 Bootstrap samples). The results indicated no significant differences in the key paths, as detailed in the table below: CA→EP: Δβ=0.006, p>0.05; CA → ESE: Δβ=0.049, p>0.05; EP → ESE: Δβ=0.081, p>0.05; EP → EI: Δβ=0.027, p>0.05; ESE → EI: Δβ=0.024, p>0.05. The data results suggest that there are no significant differences in the key paths; see Table 3 for details.

We have included a verification of this section in the results portion of the article; please refer to lines 525-536.

**Table 3** Intergroup difference testing of gender in the main path of the model

| **Path** | **Female Standardized Regression Coefficients** | **Male Standardized Regression Coefficients** | **Significance of Group Difference** |
| --- | --- | --- | --- |
| **CA → EP** | 0.812 | 0.806 | Δβ=0.006, p=0.901 |
| **CA → ESE** | 0.561 | 0.610 | Δβ=0.049, p=0.423 |
| **EP → ESE** | 0.286 | 0.367 | Δβ=0.081, p=0.067 |
| **EP → EI** | 0.601 | 0.628 | Δβ=0.027, p=0.682 |
| **ESE → EI** | 0.314 | 0.338 | Δβ=0.024, p=0.725 |

3. Theoretical Basis

This study concentrates on psychological mechanisms, (uch as cognitive-affective mediating pathways, rather than demographic differences. Meta-analytic evidence suggests that gender factors contribute to less than 3% of the explained variance in entrepreneurial intention predictors (Schlaegel & Koenig, 2014, Journal of Business Venturing), aligning with the findings of this research. "The study's sample exhibits a significant gender imbalance, with 80% being female. Although additional analyses suggest that the core mechanisms are stable across genders, future research must validate these pathways in more balanced samples—particularly in certain cultural contexts where the development of entrepreneurial self-efficacy may vary by gender (Marlow & McAdam, 2013)." This section has been appended to the limitations of the article; please refer to lines 868 to 872.

Schlaegel, C., & Koenig, M. (2014). Determinants of entrepreneurial intent: A meta–analytic test and integration of competing models. *Entrepreneurship theory and practice*, *38*(2), 291-332.

Marlow, S., & McAdam, M. (2013). Gender and entrepreneurship: Advancing debate and challenging myths; exploring the mystery of the under‐performing female entrepreneur. *International Journal of Entrepreneurial Behavior & Research*, *19*(1), 114-124.

**Q3.2:**- The sample is limited to three institutions in one province, which undermines claims of broad applicability or international relevance.

***Reply3.2:***

Thank you for your valuable comments. In response to your concern, we have revised the manuscript to clarify the rationale behind our sampling strategy and to contextualize the findings’ applicability. The key points are as follows:

1. We have added a theoretical basis in section 3.2 to explain the rationale and representativeness of the sample; please refer to lines 345-359.

2. We have supplemented the discussion section with the reasons for sample selection; please refer to lines 547-565.

3. In the "Limitations and Future Research Directions" section, we have included the limitations of the sample; please refer to lines 843-867.

4. We have made wording adjustments in the "Introduction" section; please refer to lines 143-145.

**Q4:** In Section 4 “Results”

The claim of full mediation is contradictory. If the total effect is significant (β = 0.669), but the direct effect is non-significant, a partial mediation model is more likely than full mediation unless the indirect effects explain the entire variance.

***Reply4:***Thank you for your valuable comments. Our explanation is as follows, with corresponding supplementary notes in the text, specifically on lines 517-521. The details are as follows:

The mediation effect can be divided into complete mediation and partial mediation. Complete mediation refers to the situation where the independent variable's effect on the dependent variable is entirely transmitted through the mediator variable, with no direct effect. Partial mediation indicates that there are both direct and indirect effects. According to the classic mediation analysis framework (Baron & Kenny, 1986), if the total effect is significant but the direct effect is not significant, while the indirect effect is significant, it typically supports a complete mediation model (i.e., the independent variable's effect on the dependent variable is fully transmitted through the mediator variable).

In this study,The mediation effect test results for EP show that the upper and lower limits of the 95% confidence interval do not include 0, indicating that EP plays a mediating role in the impact of CA on EI, with a mediation effect of 0.479, accounting for 71.6% of the total effect. The indirect effect of the CA-EP-EI path accounts for the vast majority of the total effect (71.6%), indicating that the mediating role of entrepreneurial passion plays a crucial part in the influence of career adaptability on entrepreneurial intention. Hypothesis 2 is supported. The mediation effect test results for ESE show that ESE mediates the impact of CA on EI, with a mediation effect of 0.181, accounting for 27.1% of the total effect.The indirect effect of the CA-ESE-EI path accounts for 27.1% of the total effect. The illustrates that entrepreneurial self-efficacy plays a mediating role in the impact of career adaptability on entrepreneurial intention. Hypothesis 3 is supported. The test results for the chain mediation effect of EP and ESE show that the serial mediation effect is significant, with a mediation effect of 0.075, accounting for 11.2% of the total effect. The indirect effect of the CA-EP-ESE-EI path accounts for 11.2% of the total effect. This indicates that entrepreneurial passion and entrepreneurial self-efficacy play a chain mediation role in the influence of career adaptability on entrepreneurial intention (see Table 3). Hypothesis4 is supported.

Given the significant total effect of the aforementioned chain mediation model (β=0.669, 95% CI [0.000, 0.601]), and the non-significant direct effect (β=-0.066, 95% CI [-0.142, 0.84]), the significant indirect effect explains all the variance, suggesting a complete mediation effect (Hayes, 2022). Numerous academic studies have also utilized this determination method and standard (Qiu et al., 2023; Xiao et al., 2024).

Baron, R. M., & Kenny, D. A. (1986). The moderator–mediator variable distinction in social psychological research: Conceptual, strategic, and statistical considerations. Journal of Personality and Social Psychology, 51(6), 1173–1182. [https://doi.org/10.1037/0022-3514.51.6.1173](https://doi.org/10.1037/0022-3514.51.6.1173)

Hayes, A. F. (2022). Introduction to mediation, moderation, and conditional process analysis: A regression-based approach (3rd ed.). Guilford Press.

Qiu, W. F., Ma, J. P., Xie, Z. Y., Xie, X. T., Wang, C. X., & Ye, Y. D. (2023). Online risky behavior and sleep quality among Chinese college students: The chain mediating role of rumination and anxiety. *Current psychology*, *42*(16), 13658-13668.

Xiao, T., Pan, M., Xiao, X., & Liu, Y. (2024). The relationship between physical activity and sleep disorders in adolescents: a chain-mediated model of anxiety and mobile phone dependence. *BMC psychology, 12*(1), 751.

**Q5:** In Section 5 “Discussion”

- The discussion repeats descriptive findings and lacks depth in theorizing unexpected or context-specific outcomes.

- Cultural explanations (e.g., Confucian values, collectivism) are absent, despite the exclusive Chinese context.

***Reply5:***Thank you for your valuable comments. We have rewritten this section (542-827), particularly adding cultural explanations (e.g., Confucian values, collectivism); please see lines 661-692.

**Q6:** Language and Style

**Q6.1:**+ The manuscript still contains awkward phrasing and occasional grammatical errors. For example:

- “This study has been ethically reviewed and approved in writing...” >> could be more concise: “The study received written ethical approval.”

- “This phenomenon might be due to...” >> revise to: “This result may stem from...”

***Reply6.1:***Thank you for your valuable comments. We have retranslated the text and utilized language editing services to enhance its fluency and accuracy. Based on your suggestions, we have specifically revised the two sections you mentioned, lines 331-332 and 570-572.

**Q6.2：**Language fluency needs improvement in several parts. Some phrasing is awkward or unidiomatic.

- Figures and tables are dense and difficult to interpret. Some regression tables lack clarity (e.g., missing SE, unclear headings).

- APA 7 formatting is inconsistent.

>> Suggest proofreading the manuscript for smoother academic English, possibly with a language editing service.

***Reply6.2:***

Thank you for your valuable comments. We have revised the paper according to your suggestions. The details are as follows:

1.The tables have been reorganized, and the regression table has been updated to include standard errors (SE). The modified tables can be found in lines 496-499.

2.Regarding the format of references, while most journals adhere to APA7, the journal to which we have submitted our work, PLOS ONE, has its own specific formatting requirements. Consequently, we need to use the PLOS ONE reference style, not the APA7 format. For further details, please consult the journal's websit: <https://journals.plos.org/plosone/s/submission-guidelines#loc-references>e.

3. The entire text has undergone language editing services to enhance its fluency and accuracy.

The above is our complete response. Thank you for your review. Your suggestion enhances the perfection of our paper.
