# Supplementary material for: How career adaptability affects entrepreneurial intention: The chain-mediated roles of entrepreneurial passion and self-efficacy
Source: PLoS One. 2025 Sep 2;20(9):e0329651. doi: 10.1371/journal.pone.0329651 (PMC12404465; doi:10.1371/journal.pone.0329651)
Supplement: Attachment — Submitted filename: [2025.7.17]Response to Reviewers.docx [file pone.0329651.s004.docx]

Dear Editor:

It is our big honor to have you be our editor, and we appreciate your attention and recognition for our manuscript. Many thanks to reviewers for giving us a lot of valuable suggestions. Based on the suggestions of the reviewers, we have made a lot of revisions to the manuscript, and we are confident to submit a high-quality manuscript. Below are our responses based on the comments of the reviewers, thank you again.

**To** **reviewers 3:**

Dear reviewer 3:

Thank you very much for your time involved in reviewing the manuscript and your comments have further improved the quality of the manuscript. We have carefully reviewed the comments and revised the manuscript accordingly. Hope the explanation has fully addressed all of your concerns. Point-by-point responses to reviewers are as follows:

**Q1:** The introduction, while improved, highlight more clearly what this model adds beyond previous chain mediation models in career development and entrepreneurship.

***Reply1:***Thank you for your valuable comments. We have added this section in the introduction; please see lines 143-156.

**Q2:** In Section 2. “Literature review and research hypothesis”, remaining concerns:

The theoretical novelty remains limited. SCT is a widely used framework, and its application here does not significantly advance existing theoretical models. The classification of constructs into SCT categories (e.g., CA as an environmental factor) appears oversimplified and conceptually ambiguous. Career Adaptability is arguably more trait-like and individual-level than environmental.

***Reply2:***Thank you for your valuable comments. We have made revisions regarding this issue, including the introduction, literature review, and discussion sections. Please see lines 92-99, 119-123, 258-280, 321-323, 589-596, and 659-661.

The above is our complete response. Thank you for your review. Your suggestion enhances the perfection of our paper.
